# Retraction notice for: “Baicalein restrains proliferation, migration, and invasion of human malignant melanoma cells by down-regulating colon cancer associated transcript-1” [Braz J Med Biol Res (2019) 52(12): e8934]

**DOI:** 10.1590/1414-431X2020e8934retraction

**Published:** 2021-07-23

Xiaoliang Yang^1^ https://orcid.org/0000-0002-8789-3168, Jinjie Jiang^2^ https://orcid.org/0000-0002-4934-8281, Chunyan Zhang^1^ https://orcid.org/0000-0003-4929-7610, Yinghao Li^1^ https://orcid.org/0000-0001-9300-4569

^1^Department of Burn and Plastic Surgery, Qingdao Central Hospital, The Affiliated Central Hospital of Qingdao University, Qingdao, Shandong, China

^2^Department of Traditional Chinese Medicine, Qingdao Central Hospital, The Affiliated Central Hospital of Qingdao University, Qingdao, Shandong, China

**Retraction for:** Braz J Med Biol Res | doi: 10.1590/1414-431X20198934 | PMID: 31778440 | PMCID: PMC6886380

The authors would like to retract the article “Baicalein restrains proliferation, migration, and invasion of human malignant melanoma cells by down-regulating colon cancer associated transcript-1” that was published in volume 52 no. 12 (2019) (Epub Nov 25, 2019) of the Brazilian Journal of Medical and Biological Research.

After the publication of this study, the corresponding author requested its retraction due to “the identification of unspecified data inconsistency that could lead to mistaken conclusions.” The Editors agreed with and endorsed that decision.

The Brazilian Journal of Medical and Biological Research had received authorization from all authors before the publication of the paper. We regret the unprofessional behavior of the authors involved.

Correspondence: Yinghao Li: <li06yh@126.com>

